# Potential Application of h-BNC Structures in SERS and SEHRS Spectroscopies: A Theoretical Perspective

**DOI:** 10.3390/s19081896

**Published:** 2019-04-21

**Authors:** Sara Gil-Guerrero, Nicolás Otero, Marta Queizán, Marcos Mandado Alonso

**Affiliations:** Department of Physical Chemistry, University of Vigo, Lagoas-Marcosende s/n, 36310 Vigo, Spain; sgg@uvigo.es (S.G.-G.); nom05@uvigo.es (N.O.); mqueizan@uvigo.es (M.Q.)

**Keywords:** SERS, SEHRS, h-BNC, graphene, polarizability, hyperpolarizability, quantum modelling

## Abstract

In this work, the electronic and optical properties of hybrid boron-nitrogen-carbon structures (h-BNCs) with embedded graphene nanodisks are investigated. Their molecular affinity is explored using pyridine as model system and comparing the results with the corresponding isolated graphene nanodisks. Time-dependent density functional theory (TDDFT) analysis of the electronic excited states was performed in the complexes in order to characterize possible surface and charge transfer resonances in the UV region. Static and dynamic (hyper)polarizabilities were calculated with coupled-perturbed Kohn-Sham theory (CPKS) and the linear and nonlinear optical responses of the complexes were analyzed in detail using laser excitation wavelengths available for (Hyper)Raman experiments and near-to-resonance excitation wavelengths. Enhancement factors around 10^3^ and 10^8^ were found for the polarizability and first order hyperpolarizability, respectively. The quantum chemical simulations performed in this work point out that nanographenes embedded within hybrid h-BNC structures may serve as good platforms for enhancing the (Hyper)Raman activity of organic molecules immobilized on their surfaces and for being employed as substrates in surface enhanced (Hyper)Raman scattering (SERS and SEHRS). Besides the better selectivity and improved signal-to-noise ratio of pristine graphene with respect to metallic surfaces, the confinement of the optical response in these hybrid h-BNC systems leads to strong localized surface resonances in the UV region. Matching these resonances with laser excitation wavelengths would solve the problem of the small enhancement factors reported in Raman experiments using pristine graphene. This may be achieved by tuning the size/shape of the embedded nanographene structure.

## 1. Introduction

Since the discovery of the Surface Enhanced Raman Scattering (SERS) activity of extended graphene sheets [1], also known as Graphene Enhanced Raman Scattering (GERS) phenomena [2,3,4], experimental and theoretical efforts have been devoted to elucidate the possible enhancement mechanisms [5,6,7,8,9,10,11,12,13]. It is well-known that the closeness of the energy gap in extended graphene structures makes the optical activity and plasmon emission to be located within the THz region [14], ruling out the possibility of electromagnetic enhancement using UV laser sources. Thus, a strong enlargement of the Raman activities of target molecules adsorbed on a graphene sheet is *a priori* unexpected. The experiments confirmed the small enhancement factor [2,3,4], reaching two orders of magnitude at most, and its origin in charge transfer (CT) transitions between the surface and the adsorbed molecule [1,6,10,12]. Even though a chemical mechanism based on charge transfer excitations could yield larger enhancement factors (EF), it is less likely that these factors might reach the Raman enhancements obtained with metallic nanoparticles, which were reported to be larger than ten orders of magnitude [15].

However, other advantages with respect to metallic nanoparticles arise using graphene as SERS platform. Among them, three can be remarked: the improved signal-to-noise ratio due to an efficient quenching of the fluorescence in the adsorbed molecule [16,17], leading to detection limits of 8 × 10^−10^ M [2] for rhodamine-6G (R6G), which are similar to those found for metallic structures; a better selectivity caused by the noncovalent nature of the molecule-substrate interaction [18]; and the economic benefits according to its lower production cost with respect to more sophisticated nanofabrication techniques. Such techniques are employed to control aggregation of the colloidal system and the spatial arrangement of the metallic nanoparticles, which is a requirement to obtain reproducible results in SERS [19]. In fact, promising chemical sensors based on GERS have been recently proposed for protein detection in blood [20] and for the detection of fluorescent organic molecules [21].

In search of an improvement in the sensibility of GERS, the effects of surface doping have been also explored. Thus, nitrogen doping of the graphene surface was found to increase the enhancement factors in organic dyes due to the better alignment of the surface Fermi level with the dyes’ highest occupied molecular orbital (HOMO) and lowest occupied molecular orbital (LUMO) [9,21,22,23], reaching detection limits of 5 × 10^−11^ M for R6G [21]. Nevertheless, the enhancement is still low compared to metallic platforms, since it still relies on the efficiency of charge transfer excitations. Another strategy to improve the Raman enhancement ability of graphene structures would be shifting the optical response by making use of nanostructures instead of extended surfaces. Recent theoretical works demonstrated that certain carbon nanostructures may display electronic transitions with plasmonic features in the UV range [24,25], suggesting the possibility of using large- and medium-size polycyclic aromatic hydrocarbons to enhance the Raman activity of smaller molecules attached to their surfaces. Such possibility was explored recently by some of us on different carbon allotropes, including graphene nanodisks, fullerenes and nanotubes, with time-dependent density functional theory (TDDFT) [26,27]. The following important conclusions were extracted from these works regarding the Raman enhancement mechanism by carbon surfaces: when approaching resonance, CT may give rise to enhancement factors comparable to resonance Raman mechanisms; CT occurs for large molecule-surface contacts, such as in stacking interactions; surface resonance effects in the carbon structure may lead to huge enhancement of the Raman activity of the adsorbed molecule favored by small molecule-surface vibrational couplings. In addition, it is worth to mention that the influence of CT in GERS was also theoretically explored using even simpler models for carbon structures [28] and gold-graphene models [29].

The main drawback for the implementation of nanographenes into Raman-based sensors is the immobilization of the ‘nanographene + molecule’ complex. This may be done by deposition on SiO_2_/Si surfaces, metallic particles or even graphene sheets. However, it would be desirable to have the nanographene structure embedded in an extended surface, so that only the physicochemical adsorption process of the analyte would take place. By boron-nitrogen (BN) doping of a graphene sheet, graphene nanoislands can be created within the extended surface, which, due to the strong π-electron confinement exerted by the BN sections, keep similar π-electron delocalization patterns as the corresponding isolated nanographenes [30,31]. The optical response properties of these structures have been investigated recently using quantum chemical methods [32,33,34]. These BN-doped carbon surfaces are known as h-BNC structures. Thanks to the recent progress in BN-doping experimental techniques [35,36,37] one may predict that very different morphologies for the embedded nanographene structures will be possible. These techniques involve from processes where doping takes place during the growth of the graphene surface [35] to processes where a direct substitution of C-C by B-N units is performed in a pristine graphene sheet [36].

In this work, we analyze, using state-of-the-art quantum chemical methods, the suitability of these embedded nanographene structures as potential platforms for optical spectroscopic techniques based on Raman and Hyper-Raman enhancement effects. Herein, we first focus on the electronic structure and molecular affinity of the embedded nanographenes in comparison with the corresponding isolated structures. For the adsorption study, the pyridine molecule is employed as model system. Then, using typical UV laser sources, we evaluate the frequency dependent electric dipole polarizabilities and first-order hyperpolarizabilities, including local effects. These properties are at the heart of the calculation of Raman and Hyper-Raman activities, so that they are crucial to understand the enhancement mechanisms. Large molecular affinities, similar or even larger than those previously reported for isolated nanographenes, are found for the h-BNC structures investigated, mainly due to the formation of strong dispersive forces. Enhancement factors around 10^3^ and 10^8^ are obtained for the polarizability and first order hyperpolarizability, respectively, when the excitation wavelength approaches that corresponding to strong electronic transitions located at the embedded nanographene structures. This points out that nanographenes embedded within hybrid h-BNC structures may be employed as platforms for enhancing the (Hyper)Raman activity of molecules immobilized on their surfaces.

## 2. Methodology

**Figure 1:**
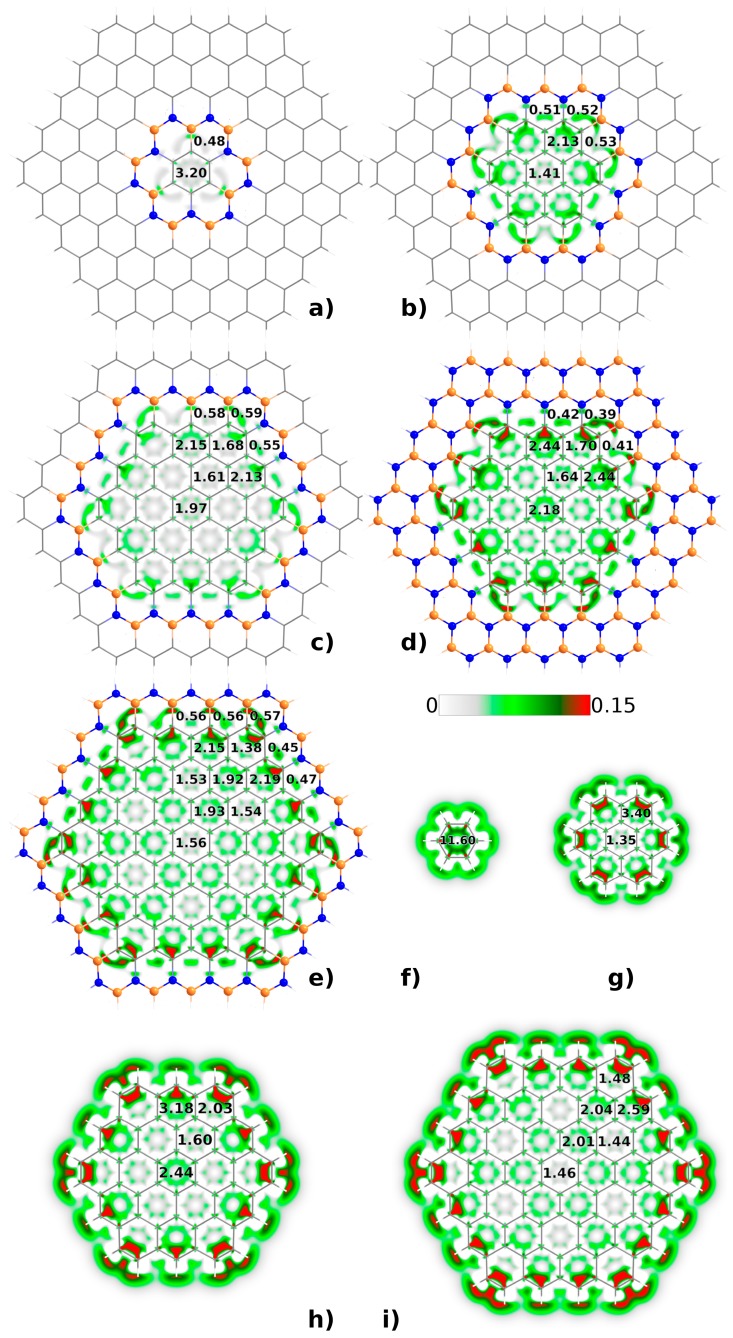
h-BNCs and isolated nanographene structures investigated in this work. Orange and blue atoms correspond to boron and bitrogen, respectively. C6BN (**a**), C24BN (**b**), C54BN (**c**), C54BN2 (**d**), C96BN (**e**), C6 (**f**), C24 (**g**), C54 (**h**) and C96 (**i**). 6-center delocalization indices for the nonequivalent benzenoid rings (multiplied by 10^3^) are shown in the figure as well as the intrinsic polarizability density.

The h-BNC structures considered for this work can be viewed in Figure 1. They offer a compromise between the computational demands of large systems and a sufficient surface extension in order to contain embedded nanographenes of proper size. Thus, we have selected h-BNCs including up to one hundred and fifty atoms, excluding the peripheral hydrogens added to saturate the structure. 

**Figure 2:**
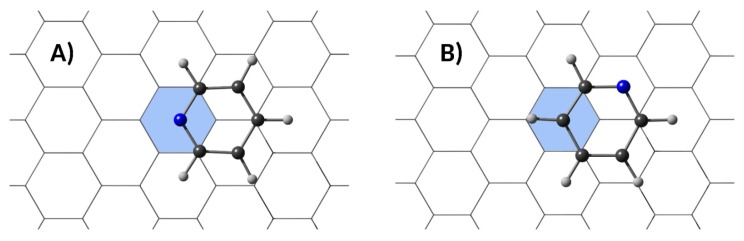
The two stable stacking dispositions of the pyridine molecule with respect to the carbon nanodisks. The colored ring represents the central ring of the nanodisk. (**A**) and (**B**) correspond to C_S_ and C_1_ group symmetries, respectively.

In the inner shell of these h-BNCs, nanographenes containing up to ninety-six carbon atoms can be created. As mentioned in the previous section, pyridine was employed as model system to analyze the molecular affinities of the embedded nanostructures. An exploration of the potential energy surface showed that the two most stable complexes correspond to stacking conformations. The difference stems from the orientation of the pyridine molecule with respect to the B-N string. The global structure possesses C_S_ or C_1_ symmetry depending on which atom from the pyridine ring, nitrogen or carbon, is centered in the central ring of the h-BNC (see the representation of Figure 2). The results obtained for the h-BNCs will be contrasted with the results obtained for the isolated carbon nanodisks.

Electronic structure calculations on h-BNC structures and complexes formed with pyridine were performed using density functional theory (DFT) with the M062X-D functional and the 6-31G(d) basis set. This functional was found to perform much better for pyridine + graphene complexes than other hybrid functionals like B97-D, BLYP-D, B3LYP-D and wB97XD, requiring a much smaller empirical dispersion correction and approaching the complex deformation densities to those obtained by post-Hartree-Fock methods like Coupled Cluster with single and double excitations (CCSD) or second order Møller-Plesset (MP2) [38]. Basis set superposition error was corrected with the counterpoise method [39]. Calculations of total interaction energies and complex and monomers’ electron densities were carried out with the Gaussian 09 program package [40]. The interaction energy decomposition analysis was performed using an own Fortran code. The interaction energy fragmentation scheme employed in this work is based on the splitting of the one- and two-electron densities into isolated monomers’ densities and interaction terms [38,41]. The interaction energy is decomposed into electrostatic, repulsion, exchange, induction and rest of polarization (mainly dispersion) according to Equation (1) (for details see references [38,41]):

(1) $$E_{Int}=E_{Elec}+E_{Rep}+E_{Exch}+E_{Ind}+E_{Res\text{-}Pol}$$

An empirical dispersion correction may be obtained separately, so that it must be added to the *E_Res-Pol_* term to get the total interaction energy. This energy decomposition scheme allows for the calculation of the deformation electron densities associated to the Pauli (*E_Rep_* + *E_Exch_*) and Polarization (*E_Ind_* + *E_Res-Pol_*) components separately, which is useful for further visualization of the interaction strength and for comparing between embedded and isolated nanographenes. These deformation densities were represented using the Chemcraft visualization program [42]. The empirical dispersion corrections of Grimme et al. were computed with the DFT-D3 program [43].

Analysis of the different electronic resonances in the h-BNCs was carried out using time-dependent DFT (TDDFT) with the same functional and basis set. Comparison with *a priori* more appropriate functionals for excited states, like CAM-B3LYP, reflected no significant differences in our systems. Thus, to be consistent with the rest of the work, we will discuss here the results obtained with M062X. 

Polarizability, *α*, and first-order hyperpolarizability, *β*, static and dynamic, were calculated using the coupled-perturbed Kohn-Sham (CPKS) approach with the Gaussian09 program. The same program was employed to extract the necessary density derivatives to partition the polarizability tensor components, *α_ij_*, into intrinsic atomic terms according to Equation (2) [44]:

(2) $$\alpha_{ij}^{A}=\int w_{A}\left(\vec{r}\right)\left(r^{i}-R_{A}^{i}\right)\left(\frac{\partial \rho \left(\vec{r}\right)}{\partial \varepsilon^{j}}\right)d\vec{r}$$

where *w_A_* represents the weight factor for atom *A* at point *r*, *r_i_* and *R_A_^i^* are the *x*, *y* or *z* components of the electron and nuclear positions, respectively, and *ρ* and *ε^j^* are the electron density and the *x*, *y* or *z* components of a constant electric field, respectively. From the integrand of Equation (2) one can further define the intrinsic polarizability density according to Equation (3) [45]:

(3) $$\alpha_{ij}^{}\left(\vec{r}\right)=\sum_{A}w_{A}\left(\vec{r}\right)\left(r^{i}-R_{A}^{i}\right)\left(\frac{\partial \rho \left(\vec{r}\right)}{\partial \varepsilon^{j}}\right)$$

Equation (3) allows constructing real space intrinsic polarizability plots, which in this work were obtained with the POLACUBE program (available upon request).

Finally, electron delocalization patterns within the benzenoid rings of the nanographenes were obtained using multicenter electron delocalization indices [46,47,48,49,50]. These indices measure the degree of multicenter electron delocalization within a given set of *n* atoms from the integration of the *n*-order electron density within the corresponding atomic domains. In this work, we have defined the atomic domains, also for the calculation of intrinsic polarizabilities, using the Hirshfeld iterative method (H-I) [51]. Thus, Equation (4) represents the *n*-center delocalization index with the H-I atomic partitioning for a closed-shell molecule:

(4) $$\Delta_{n}=2\sum_{I=1}^{n!}{\hat{P}}_{I}\left[\sum_{i}^{N_{occ}^{}}\sum_{j}^{N_{occ}^{}}\sum_{k}^{N_{occ}^{}}\ldots \sum_{m}^{N_{occ}^{}}\int w_{A}(\vec{r})\phi_{i}(\vec{r})\phi_{j}(\vec{r})d\vec{r}\int w_{B}(\vec{r})\phi_{j}(\vec{r})\phi_{k}(\vec{r})d\vec{r}\ldots \int w_{M}(\vec{r})\phi_{m}(\vec{r})\phi_{i}(\vec{r})d\vec{r}\right]$$

in which capital letters refer to the *n* atoms A, B, …, M, *ϕ* refers to doubly occupied molecular orbitals and *P_I_* is the permutation operator that takes into account all possible *n*! permutations of the atomic labels. Multicenter delocalization indices were calculated using the NDELOC program (available upon request).

## 3. Results and Discussion

### 3.1. Electronic Properties of h-BNCs vs Isolated Graphene Nanodisks

Among the electronic features relevant for this work, we are mainly interested in the electron delocalization patterns and electric polarizabilities. Both are intrinsically related to the optical response and molecular affinity. The results obtained for the six-center delocalization indices of the nonequivalent benzenoid rings in the h-BNCs studied are incorporated to Figure 1. We have calculated the same indices in the isolated nanodisks for the sake of comparison, which are also included in Figure 1. As can be observed, the π-electron delocalization patterns are similar in isolated and embedded nanostructures, alternating Kekulé and Clar patterns when going from smaller (C24) to larger (C96) nanodisks. This reflects an important degree of confinement of the π-electron system in the h-BNCs exerted by the surrounding B-N strings. However, even though the patterns are similar, the multicenter indices of the outer rings differ significantly in embedded and isolated nanographenes. They are clearly smaller in the h-BNCs and the explanation can be found in the extension of the π-electron delocalization from the edges of the nanodisk to the B-N string. However, this extension is certainly small and we can argue that π systems at the two sides of the B-N frontier are quasi-independent each other. On one hand, if outer and inner π systems were effectively “connected”, then the delocalization patterns in the embedded nanographenes should differ with respect to the isolated ones. On the other hand, if the B-N string were contributing significantly to the π-electron delocalization of the outer and inner systems, then C_3_NB_2_ and C_3_N_2_B rings should display a large multicenter index. Therefore, we can conclude that the inner and outer π-electron structures are delocalized almost independently of one another.

Particularly interesting is the comparison between the circumcoronene nanodisks in C54BN and C54BN2, the latter with a surrounding string of borazine rings instead of a single thread of boron and nitrogen atoms. As regards to the π-electron delocalization, the values of multicenter delocalization indices in C54BN2 are closer to those obtained for the isolated circumcoronene, reflecting a larger degree of π-electron confinement in C54BN2 than in C54BN. Thus, as expected, the degree of π-electron confinement increases with the number of threads in the surrounding B-N string.

The intrinsic polarizability density plots within the region of the carbon nanodisks are also shown in Figure 1. The plots reflect similar polarizability distributions in embedded and isolated nanographene structures of the same size. The radically different nature of the C-H and C-N/C-B σ bonds makes the peripheral region of the nanodisks be different too. Table 1 also reflects a comparison between isolated and embedded nanographenes. In this case, the integrated values of the intrinsic polarizability function within the corresponding nanodisks are shown. As can be seen, large differences are reported for the smallest C6BN structure with respect to the isolated benzene ring. However, the differences are drastically reduced with the size of the nanographene structure and with the effectiveness of the π-electron confinement. Thus, the ratio between the values of isolated and embedded nanodisks goes to 1, following the order C6BN >> C24BN > C54BN > C54BN2 ≈ C96BN.

According to the previous discussion, it is expected that the “pure” chemical effects for nanodisks presenting a significant size with respect to the adsorbed molecule, in our case for C54 and C96, do not show significant differences between embedded and isolated platforms. On the contrary, resonance effects may occur at different wavelengths due to the effect exerted by the peripheral bonding on the occupied-virtual orbital energy gaps. This issue will be discussed later.

### 3.2. Stability of Pyridine Adsorbed on Embedded Nanographenes

**Figure 3:**
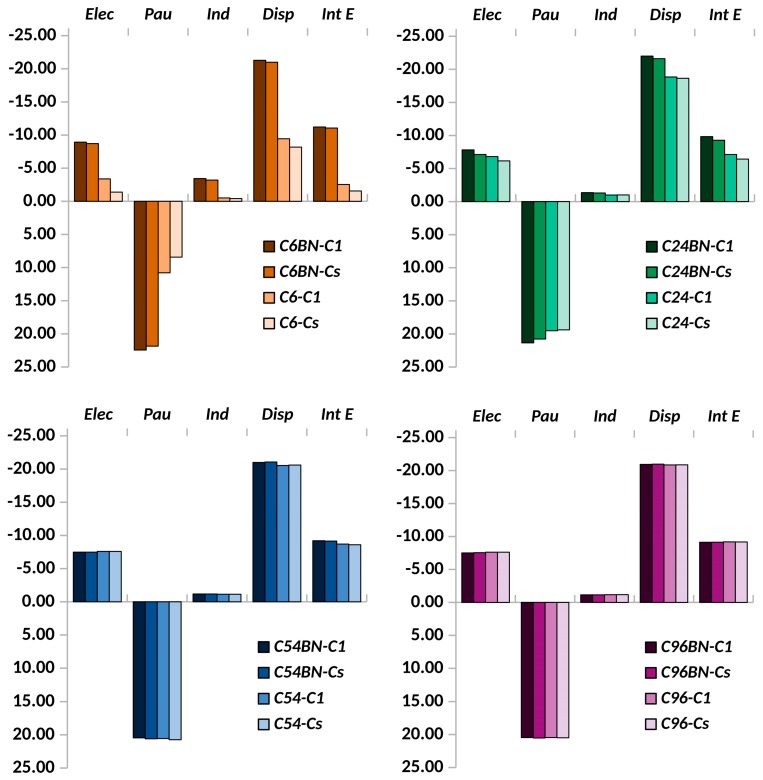
Comparison of the interaction energy (*Int E*) and its components, electrostatic (*Elec*), Pauli repulsion (*Pau*), induction (*Ind*) and dispersion (*Disp*), for the complexes formed by pyridine molecule and the embedded and isolated carbon nanodisks.

As mentioned in the previous section, two different stacking dispositions of the pyridine molecule over the nanographene surface were found to be stable. In Figure 3, the total interaction energies and their components (see Equation (1)) are compared for embedded and isolated nanographenes of different size. In this section, we will not consider the C54BN2 structure since it contains the same nanodisk as C54BN. Differences in interaction energies between both structures were found to be negligible. Even though the stability of C_S_ and C_1_ conformations is quite similar, the most stable one always corresponds to C_1_. Significant differences are observed in the smaller graphene nanodisks (C6BN and C24BN) whereas they are almost negligible in the larger ones (C54BN and C96BN). The relative weight of each component keeps almost constant along the series, with the exception of C6BN, which, due to the proximity of the pyridine molecule to the B-N string, shows larger contributions of electrostatic and induction terms. Thus, the large polarity of the B-N bonds favors the conformation where the pyridine nitrogen is orientated towards them, increasing slightly the contribution of components where the electrostatic fields play a pivotal role.

When comparing with the isolated nanographenes, one observes pronounced differences in the smaller nanodisks but negligible differences in the larger ones. The reason is again the polarity of the B-N bonds, which enhances the electrostatic and induction terms in h-BNCs. This effect is so important that it causes an inversion in the stability order with the nanographene size. Whereas in the isolated structures the stability increases with the size of the carbon nanodisk, mainly due to the increase in the dispersion component, in h-BNCs the stability decreases with the size of the embedded carbon nanodisk. Here, it is important to note that, in h-BNCs, a given target molecule may display larger affinity by the carbon or the B-N structure depending on its polarity, bonding structure, size, etc. Therefore, a detailed adsorption study must be performed in order to interpret optical spectroscopic data in each case.

**Figure 4:**
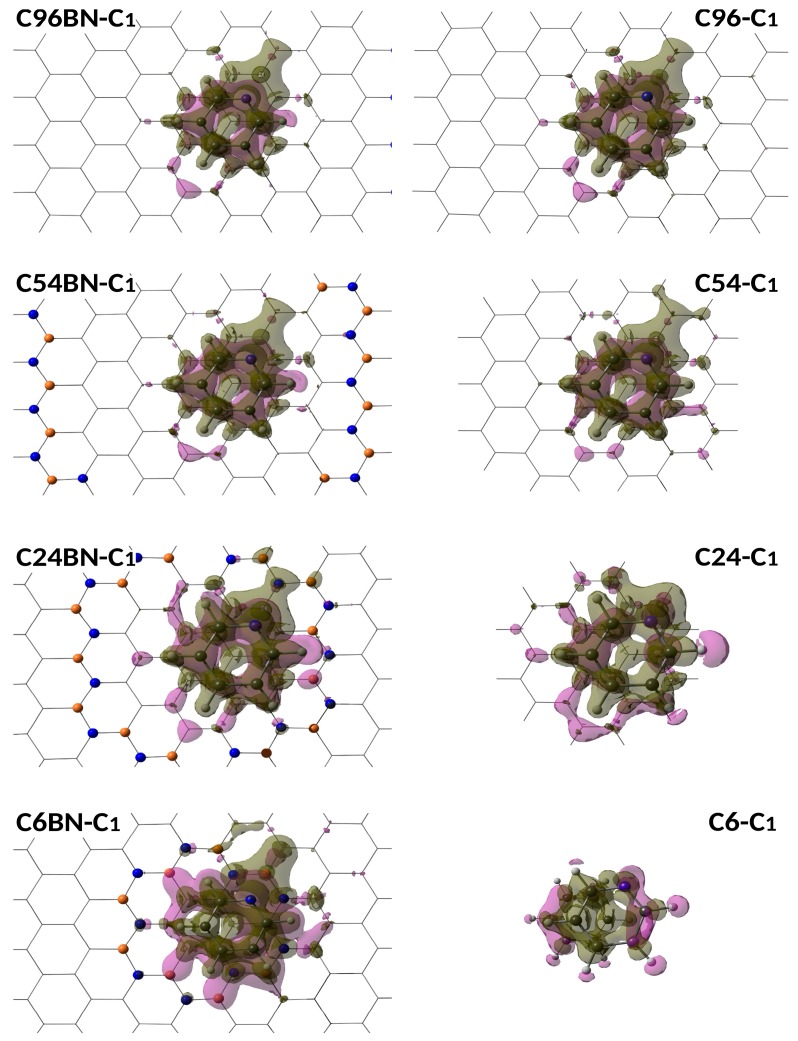
Electron polarization density obtained for the most stable complexes formed by pyridine molecule with embedded (left) and isolated (right) graphene nanodisks.

Electron deformation densities can also be employed as a similarity measure between nanodisks of different size and, in this case, between different conformations. It was established in previous works that, in order to perform a good comparison between nanographene structures, it is better to use the polarization component instead of the total deformation density. The polarization component contains the effect of dispersion energy, which is by far the most important term. In addition, electrostatic energy is not reflected on the electron deformation density since it depends on the unperturbed electron densities of the interacting systems. Figure 4 shows the polarization component of the deformation density obtained for the interaction of pyridine with the different h-BNCs. As we can observe, differences between the two conformations are appreciated only in the h-BNCs containing the smallest nanodisks (C6 and C24), the plots are almost identical for C54 and C96. The same can be said for the isolated structures, the polarization density plots are identical for the largest nanostructures, whereas significant differences are observed in the smallest ones.

### 3.3. Resonances in Pyridine+h-BNC Complexes

TDDFT calculations were carried out to study the first twenty excited electronic states in the complexes formed with the embedded nanographene structures, C24BN, C54BN, C54BN2 and C96BN. The excitation wavelength, oscillator strength values and components of the transition dipole moment for the ground-to-excited state excitations are collected in Table 2.

These data reflect only two strong quasi-degenerate resonances, which corresponds to photon adsorption in the parallel direction to the h-BNC surface (*xy* plane). The remaining excitations are much weaker according to the oscillator strength values. As can be seen, the strong resonances are reinforced (larger oscillator strength) with the size of the nanodisk. The excitation wavelength gap also narrows with the size of the nanodisk. Thus, it is expected that the oscillator strength increases and the wavelength gap reduces for even larger nanodisks, leading to a unique strong resonance that may enhance greatly the optical response of the adsorbed molecule.

**Figure 5:**
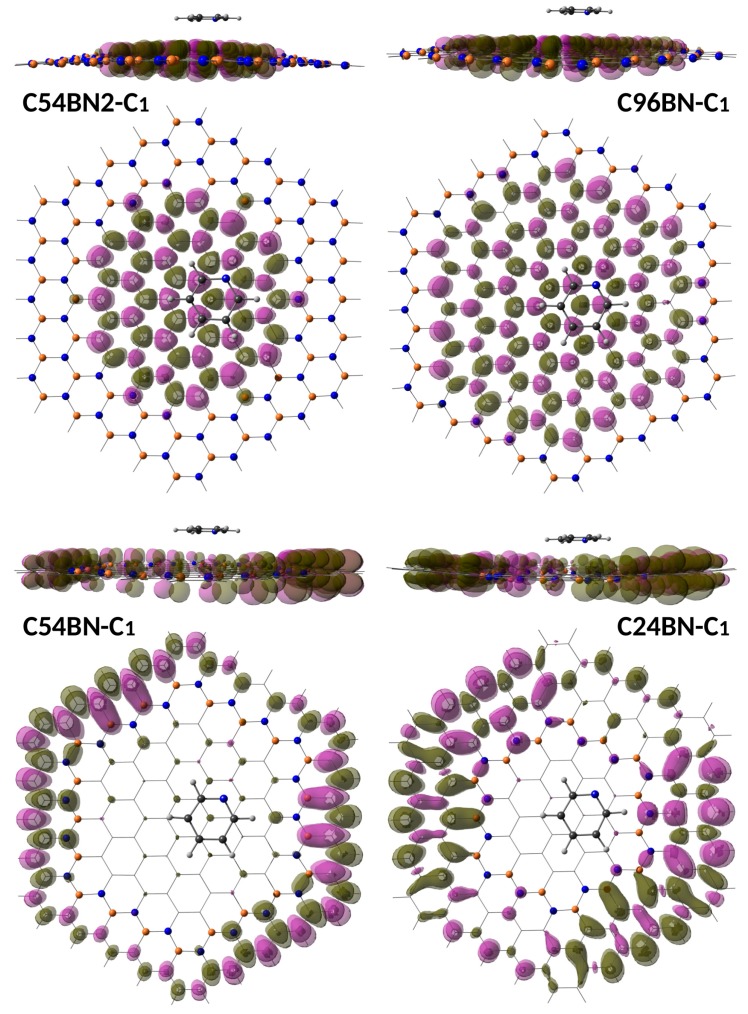
Electron density difference associated to the strongest ground-to-excited state electronic transitions in the most stable complexes formed by pyridine molecule with the h-BNCs.

In order to characterize the nature of these strong resonances, we have depicted the ground-to-excited state electron deformation densities in Figure 5. As can be observed, the electron reorganization takes places basically within the nanodisk surface in C54BN2 and C96BN, with negligible contribution at the pyridine molecule and B-N string. Thus, contrary to extended graphene sheets, these resonances do not correspond to charge transfer excitations but electronic excitations involving surface electrons. In the case of isolated graphene nanodisks and upon on-resonance conditions, surface electronic excitations were found to lead to strong Raman enhancements of adsorbed molecules such as pyridine, porphyrin or phthalocyanine [26,27]. However, the ground-to-excited state electron deformations indicate the strong resonances are not localized within the carbon nanodisk but within the outer carbon structure in C24BN and C54BN. Then, these models are not representative of a hypothetical h-BCN containing graphene nanoislands, and the analysis of the optical response will not reflect the response of the embedded nanographene structure. This is a consequence of an insufficient degree of electron confinement provided by a model containing only a single B-N string. Thus, only finite models terminated by B-N strings (C54BN2 and C96BN) are suitable for a discussion of the optical response of the embedded nanographene structure. Notice that in a hypothetical extended white graphene sheet with embedded carbon nanodisks, each of these nanodisks will not be surrounded by single or double B-N strings but multilayer B-N strings, providing a very high degree of π-electron confinement and subsequently a strong localized optical response.

### 3.4. Enhancement of (Non)Linear Response Properties in Pyridine+h-BNC Complexes

In this last subsection, we will analyze the response properties, *α* and *β*, in the complexes C24BN, C54BN, C54BN2 and C96BN. Laser excitation wavelengths frequently employed in (Hyper) Raman experiments (532 nm, 458 nm and 633 nm) and excitation wavelengths 0.3 nm redshifted with respect to the surface resonance wavelengths characterized in the previous subsection have been considered here. For the laser excitation wavelengths, we have selected those which are closer to the surface resonance wavelength in each case.

In this section we have calculated the enhancement factors, EFs (shown in Table 3), obtained for the isotropic value of *α* (*α_iso_*), and the total value of *β* (*β_T_*). These invariant quantities are given by Equations (5) and (6):

(5) $$\alpha_{iso}^{}=\frac{1}{3}\left(\alpha_{xx}+\alpha_{yy}+\alpha_{zz}\right)$$

(6) $$\beta_{T}^{}=\sqrt{\beta_{x}^{2}+\beta_{y}^{2}+\beta_{z}^{2}}$$

where each component within the square root of Equation (6) is given by:

(7) $$\beta_{i}=\frac{1}{3}\sum_{j}\left(\beta_{ijj}^{}+\beta_{jji}^{}+\beta_{jij}^{}\right)(i,j=x,y,z)$$

The EFs are obtained as the ratio between the frequency dependent and static (frequency or wavelength equal to zero) values for each excitation wavelength. The significant displacement of the laser excitation wavelengths from the surface resonance wavelengths, which is more than 20 nm in every case, makes the enhancement factor of *α_iso_* and *β_T_* be small, although not negligible, at the laser conditions. Thus, the maximum EF for the isotropic polarizability is reached using C96BN (EF = 3.36), whereas the EF for the total first-order hyperpolarizability in this case is 218.65. These EFs are not expected to lead to Raman and Hyper-Raman enhancements of the adsorbed molecule larger than 10^2^ and 10^6^, respectively.

On the contrary, using excitation wavelengths near the surface resonances, the EFs increase hugely. Thus, EFs close to 10^3^ are obtained for the isotropic polarizability using C54BN2 and C96BN. For the first-order hyperpolarizability, EFs close to 10^7^ and 10^8^ are obtained with C54BN2 and C96BN, respectively. These EFs may certainly lead to Raman and Hyper-Raman enhancements on the line of electromagnetic enhancements produced by metallic nanoparticles. In fact, this result was theoretically verified, only for Raman scattering, in isolated carbon nanodisks [26,27]. Calculations of the Raman spectra reflected EFs around 10^8^ using on-resonance conditions. Here, we prove that the same nanographene structures, but embedded in extended h-BNC sheets, lead to similar optical responses and then allow envisaging h-BNCs as potential Hyper (Raman) enhancement platforms of neighboring organic molecules.

## 4. Conclusions

In this study, the (Hyper)Raman enhancement ability of hybrid h-BNC structures containing graphene nanodisks of different sizes has been explored using quantum chemical methods. Firstly, the electronic and optical properties of the embedded nanographenes, mainly governed by the π-electron delocalization and (hyper)polarizability, were compared with the corresponding isolated (hydrogen saturated) structures. Such a comparison reflected strong similarities between embedded and isolated structures, with the same electron delocalization pattern and intrinsic polarizability distribution along the phenyl rings. The effect of the π-electron confinement exerted by the surrounding B-N string is evident using only a single thread of boron and nitrogen atoms. Addition of a second thread, so generating a string of borazine units, improves the π-electron confinement, approaching the values of the multicenter electron delocalization indices in the embedded carbon nanodisks to those obtained for the isolated ones.

Secondly, we have explored the molecular affinity of the embedded nanographenes employing pyridine as model system. For the larger graphene nanodisks, circumcoronene and circumcircumcoronene, the interaction energies as well as their components were found to be similar in embedded and isolated structures, in line with the similarities found in the electron delocalization and polarizability. Important differences were found in the smaller disks, coronene and benzene, due to the proximity of the pyridine molecule to the B-N string. Thus, the interaction energy was found to be more negative because of the participation of the highly polar B-N bonds.

Thirdly, resonances and (non)linear response properties in the complexes were analyzed with TDDFT and CPKS approaches. Strong surface resonances within the UV region were found, similar to those characterized for isolated graphene nanodisks in previous works. Using excitation wavelengths typically employed in (Hyper)Raman experiments, noticeable polarizability and first-order hyperpolarizability enhancement factors were obtained. Excitation wavelengths slightly displaced with respect to the surface resonance wavelength provoke enhancement factors around 10^3^ and 10^8^ for polarizabilities and first-order hyperpolarizabilities, respectively. All of this indicates that with a proper size/shape of the nanographene structure, strong (Hyper)Raman enhancement factors induced by resonant processes may be achieved using h-BNCs as platforms.

## Figures and Tables

**Table 1:** Total intrinsic polarizability (in au) for the graphene nanodisks depicted in Figure 1. Values obtained for the isolated nanodisks and those embedded in the h-BNCs are compared.

	Isolated	Embedded	Isolated/Embedded
**C6BN**	18.43	6.80	2.71
**C24BN**	38.12	27.72	1.38
**C54BN**	75.61	55.86	1.35
**C54BN2**	75.61	61.82	1.22
**C96BN**	124.68	104.06	1.20

**Table 2:** Excitation energies (EW) in eV, oscillator strengths (OS) and components of the transition dipole moment (dX, dY and dZ) in au, for the first twenty ground-to-excited state transitions in the most stable complexes formed by pyridine molecule with the h-BNCs.

**C24BN**	**dX**	**dY**	**dZ**	**OS**	**EW**	**C54BN**	**dX**	**dY**	**dZ**	**OS**	**EW**
1	0.00	−0.01	0.00	0.00	2.133	1	0.00	0.00	0.00	0.00	1.400
2	4.22	−0.17	−0.02	1.07	2.456	2	−5.45	0.00	0.03	1.38	1.893
3	−0.17	−4.22	0.00	1.08	2.471	3	0.00	5.44	0.00	1.38	1.900
4	−0.11	0.00	−0.06	0.00	2.683	4	−0.03	0.00	0.00	0.00	2.298
5	−0.02	0.00	0.01	0.00	2.735	5	0.00	0.04	0.00	0.00	2.344
6	−0.08	−1.24	0.00	0.10	2.774	6	0.05	0.00	−0.01	0.00	2.452
7	1.24	−0.07	0.00	0.11	2.775	7	0.00	1.42	0.00	0.12	2.472
8	−0.04	−0.96	0.00	0.07	2.858	8	1.37	0.00	−0.01	0.11	2.478
9	1.20	−0.05	−0.01	0.10	2.872	9	0.00	1.99	0.00	0.24	2.503
10	−0.16	−2.64	0.00	0.50	2.935	10	−2.14	0.00	0.01	0.28	2.510
11	2.57	−0.16	−0.01	0.48	2.937	11	0.00	0.47	0.00	0.01	2.513
12	0.01	0.11	0.00	0.00	2.944	12	0.01	0.00	−0.01	0.00	2.626
13	0.00	0.00	−0.23	0.00	2.971	13	0.00	0.05	0.00	0.00	2.634
14	0.00	−0.03	0.00	0.00	3.072	14	0.00	−0.33	0.00	0.01	2.677
15	−0.17	0.01	0.00	0.00	3.082	15	−0.37	0.00	0.00	0.01	2.683
16	0.06	0.00	−0.03	0.00	3.137	16	−0.04	0.00	0.00	0.00	2.692
17	0.00	−0.09	0.00	0.00	3.140	17	−0.36	0.00	0.00	0.01	2.847
18	−0.01	−0.45	0.00	0.02	3.210	18	0.00	0.28	0.00	0.01	2.848
19	0.42	−0.01	−0.01	0.01	3.213	19	2.46	−0.01	−0.01	0.44	2.966
20	0.00	0.01	0.00	0.00	3.266	20	−0.01	−2.54	0.00	0.47	2.966
**C54BN2**	**dX**	**dY**	**dZ**	**OS**	**EW**	**C96BN**	**dX**	**dY**	**dZ**	**OS**	**EW**
1	0.00	0.02	0.00	0.00	2.423	1	0.00	0.00	0.00	0.00	1.947
2	−0.04	0.00	0.01	0.00	2.498	2	0.06	0.00	0.00	0.00	2.002
3	−5.15	0.00	−0.02	1.94	2.986	3	−6.06	0.00	0.03	2.21	2.454
4	0.00	−5.16	0.00	1.95	2.989	4	0.00	6.08	0.00	2.22	2.456
5	0.00	0.00	0.00	0.00	3.306	5	0.00	−0.13	0.00	0.00	2.678
6	0.00	−0.45	0.00	0.02	3.340	6	0.05	0.00	−0.01	0.00	2.697
7	−0.49	0.00	−0.06	0.02	3.342	7	0.00	0.11	0.00	0.00	2.697
8	0.00	−0.30	0.00	0.01	3.470	8	0.00	−0.28	0.00	0.01	2.815
9	0.42	0.00	−0.05	0.02	3.471	9	−0.42	0.00	0.00	0.01	2.818
10	0.11	0.00	−0.03	0.00	3.691	10	0.00	−0.22	0.00	0.00	2.983
11	0.00	−0.17	0.00	0.00	3.698	11	−0.12	0.00	−0.01	0.00	3.025
12	0.00	0.18	0.00	0.00	3.800	12	0.00	0.20	0.00	0.00	3.092
13	0.17	0.00	0.09	0.00	3.808	13	0.32	0.00	−0.01	0.01	3.094
14	−0.47	0.00	−0.06	0.02	3.947	14	0.00	0.88	0.00	0.06	3.191
15	0.00	0.52	0.00	0.03	3.953	15	0.80	0.00	−0.01	0.05	3.193
16	0.02	0.00	−0.05	0.00	3.987	16	−0.10	0.00	−0.03	0.00	3.253
17	0.03	0.00	0.01	0.00	4.027	17	−2.20	0.00	0.01	0.39	3.317
18	−1.37	0.00	0.00	0.19	4.036	18	0.00	2.16	0.00	0.38	3.319
19	0.00	1.38	0.00	0.19	4.038	19	−0.02	0.00	0.01	0.00	3.408
20	0.00	0.14	0.00	0.00	4.081	20	0.00	−0.17	0.00	0.00	3.455

**Table 3:** Enhancement factors obtained for the isotropic value of *α*, *α*_iso_, and the total value of *β*, *β*_T_, using different excitation wavelengths, for the most stable complexes formed by pyridine molecule with the h-BNCs.

C24BN	*α*_iso_	*β*_T_
532.0 nm ^a^/0.0 nm	2.24	268.05
505.0 nm ^b^/0.0 nm	160	6.8 × 10^7^
**C54BN**		
633.0 nm ^a^/0.0 nm	2.07	297.64
655.3 nm ^b^/0.0 nm	300	3.9 × 10^7^
**C54BN2**		
458.0 nm ^a^/0.0 nm	1.93	43.80
415.4 nm ^b^/0.0 nm	180	0.9 × 10^7^
**C96BN**		
532.0 nm ^a^/0.0 nm	3.36	218.65
505.8 nm ^b^/0.0 nm	280	2.1 × 10^7^

^a^ Wavelengths corresponding to laser excitations; ^b^ Wavelengths corresponding to near-resonance excitations.
